# Therapeutic Effects of Traditional Chinese Exercises on Musculoskeletal Pain: A Systematic Review and Meta-Analysis

**DOI:** 10.1155/2021/5584997

**Published:** 2021-05-10

**Authors:** Zhenrui Li, Jie Zhuang, Shiwen Zhang, Qingyi He, Rui Zhao, Tursen Alima, Lei Fang

**Affiliations:** School of Rehabilitation Science, Shanghai University of Traditional Chinese Medicine, Shanghai 201203, China

## Abstract

**Background:**

The number of patients with musculoskeletal pain, which seriously affects people's quality of life, has increased. Traditional Chinese exercises are accepted and practiced to strengthen the body.

**Objective:**

This study aims to explore the efficacy of traditional Chinese exercises for the treatment of musculoskeletal pain.

**Methods:**

A comprehensive search of randomized controlled trials (RCTs) related to traditional Chinese exercises on patients with musculoskeletal pain was completed using PubMed, SinoMed, CNKI, VIP, and Wanfang Med Online databases. All RCTs published until February 2021 were considered. Two researchers independently screened the literature according to the predesigned inclusion and exclusion criteria, and data was extracted and assessed for their risk of bias via the Cochrane collaboration tool. Meta-analysis was performed using RevMan5.2 and Rx64 4.0.2 software.

**Results:**

A total of 45 RCT studies with 3178 patients were included. Traditional Chinese exercises were able to effectively alleviate patients with musculoskeletal pain (MD = −1.54, 95% confidence interval (−1.88, −1.19), *P* < 0.01). Among them, the Yi Jin Jing exercise was superior to other exercises, while Wu Qin Xi showed no significant effects. Besides, traditional Chinese exercises had significant positive effects on the dysfunction and stiffness of the waist and knee joints. Traditional Chinese exercises could effectively relieve the clinical symptoms of patients with musculoskeletal pain. Particularly, the Yi Jin Jing exercise presented the most significant positive effect on pain reduction.

## 1. Introduction

Pain is an unpleasant feeling and emotional experience related to actual or potential tissue damage [1]. It is considered the fifth vital characteristic after breathing, heartbeat, blood pressure, and pulse [2]. Notably, chronic pain has to have an increased impact on human health [3, 4]. In addition to causing physical pain, persistent pain can also cause emotional disorders such as anxiety and depression [5]. One of the most common forms of chronic pain is chronic musculoskeletal pain (CMP) [6], which is a chronic pain that occurs in soft tissues such as muscles, bones, joints, or tendons for more than three months [7, 8]. This type of persistent pain is the most common symptom of musculoskeletal system diseases [9], and it accounts for the largest proportion of persistent pain in various regions and in all age groups [10]. CMP involves more than 150 diseases of the human motor system [6], which are closely related to degenerative changes, and can lead to suffering and disability in the elderly population [11, 12]. Common CMPs include chronic low back pain, chronic osteoarthritis, osteoporosis, fibromyalgia syndrome, and myofascial pain syndrome [13]. About one-third of the world's population suffers from pain in the musculoskeletal system [14–16]. People in the age group from 45 to 64 years old have a higher incidence of CMP than people over 65 [14, 17], being the incidence in women higher than in men [12].

At present, the treatments for CMP include drug therapy, psychotherapy, and physical therapy. Drug therapy is the primary treatment, mainly using nonsteroidal anti-inflammatory analgesics, acetaminophen, tramadol, and other analgesics combined with antidepressants [13, 18]. However, there are many disadvantages in the conventional treatment model of chronic pain, such as severe side effects and poor results, causing patients to seek alternative therapies and self-regulation measures, such as acupuncture, yoga, and biofeedback therapy [19]. Studies [20–23] show that traditional Chinese exercises have shown to have good results in CMP treatment. Traditional Chinese exercises mainly include Tai Chi, Wu Qin Xi, Ba Duan Jin, and Yi Jin Jing. These exercises are widely used to prevent and treat various chronic diseases, but there is a lack of systematic meta-analysis on traditional Chinese exercises in CMP treatment. This study systematically evaluates the efficacy of traditional Chinese exercises for CMP treatment, providing evidence-based information for the clinical application of traditional Chinese exercises for CMP.

## 2. Materials and Methods

### 2.1. Search Strategy and Article Selection

The two researchers independently conducted a comprehensive search on PubMed, SinoMed, CNKI, VIP, and Wanfang Med Online databases for studies using traditional exercises to treat musculoskeletal pain until February 2021, regardless of their language. The search terms used were (“Tai Chi” OR “Ba Duan Jin exercise” OR “Yi Jin Jing exercise” OR “Wu Qin Xi exercise”) AND (“chronic low back pain” OR “knee osteoarthritis” OR “osteoporosis” OR “Fibromyalgia syndrome”). According to the characteristics of different databases, the subject words and free words were combined.

The preliminary screening was based on the title and abstract. Due to the wide range of interventions and diseases in these articles, only articles that included traditional Chinese exercises for musculoskeletal pain were considered. Two reviewers independently assessed the eligibility of these documents. When in disagreement, the two reviewers checked the full text of the article in question, and an agreement was only reached after discussion. After, an overall evaluation of the selected articles was made. Studies that met the following criteria were included in the study: (1) randomized controlled trials are included, (2) patients are adults with CMP, (3) the intervention type was by using traditional Chinese exercises, (4) peer-review publications are included, and (5) the difference between the experimental group and the control group's intervention is the use of traditional Chinese exercises, or the intervention method of the experimental group was the traditional Chinese exercises, and other therapies or standard therapies, or the intervention method of the control group was standard treatment or other therapies used in the experimental group besides the traditional Chinese exercises. If more than two groups in trials met the above criteria, the traditional Chinese exercise group was selected as the experimental group and the nonacupuncture treatment group was considered the control group for one-to-many comparisons. Trials that met any of the following criteria are excluded: (1) duplicated publications, (2) non-RCT research, (3) unavailable full text or missing data, and (4) low-quality research [24].

### 2.2. Data Extraction and Quality Appraisal

Review 5.2 software was used for literature quality evaluation and Rx64 4.0.2 for data analysis. The main result was the visual analog scale (VAS). The weighted mean difference and 95% confidence interval (CI) are used for analysis. When the mean difference between different studies was too significant, SMD was selected as the combined statistic. Significant heterogeneity between the studies was considered using the random-effects model, and *P* < 0.05 was set as a significant difference. The funnel chart was used to identify publication bias. The symmetry of the funnel chart was evaluated by bias regression analysis. When asymmetric, trimming and filling methods were used to adjust the publication bias in the meta-analysis.

## 3. Results

### 3.1. Documents Selection

**Figure 1:**
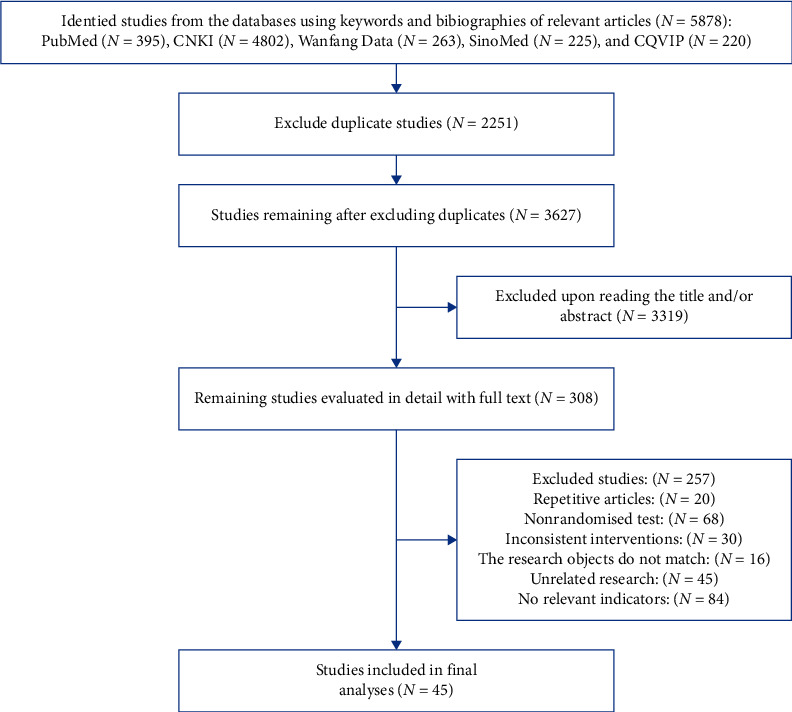
Document screening flow chart.

**Figure 2:**
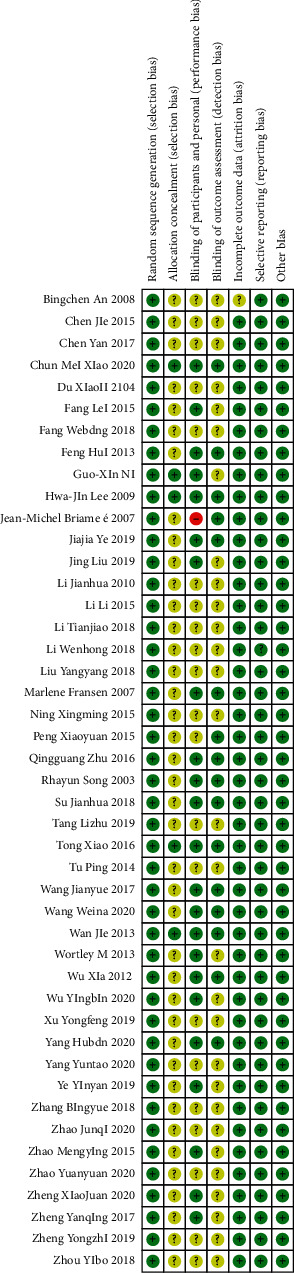
Bias risk assessment of included literature.

A total of 5878 articles were retrieved by searching the databases mentioned above. Finally, according to the inclusion and exclusion criteria, 45 studies [25–69] were included in this meta-analysis (Figure 1). All the included research intervention methods were based on traditional Chinese exercises. Among them, 14 studies [25–38] used Tai Chi, 17 studies used Ba Duan Jin exercise [39–55], eight studies [56–63] used Yi Jin Jing exercise, and six studies [64–69] used Wu Qin Xi exercise. In the same studies, 22 focused on knee osteoarthritis, 14 on low back pain, seven on osteoporosis, and two on fibromyalgia syndrome. All the included studies were randomized controlled trials. The characteristics and quality evaluation of the included studies are shown in Table 1 and Figure 2.

### 3.2. Meta-Analysis results

#### 3.2.1. Visual Analog Scale (VAS) of Pain

**Figure 3:**
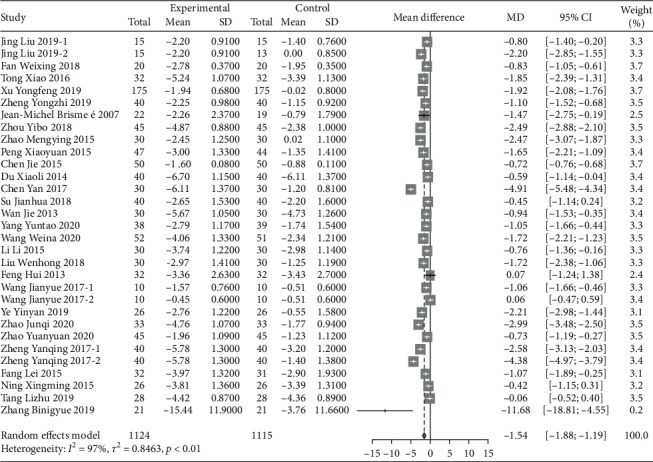
Meta-analysis of the comparison of VAS between the traditional Chinese exercise group and the control group.

**Figure 4:**
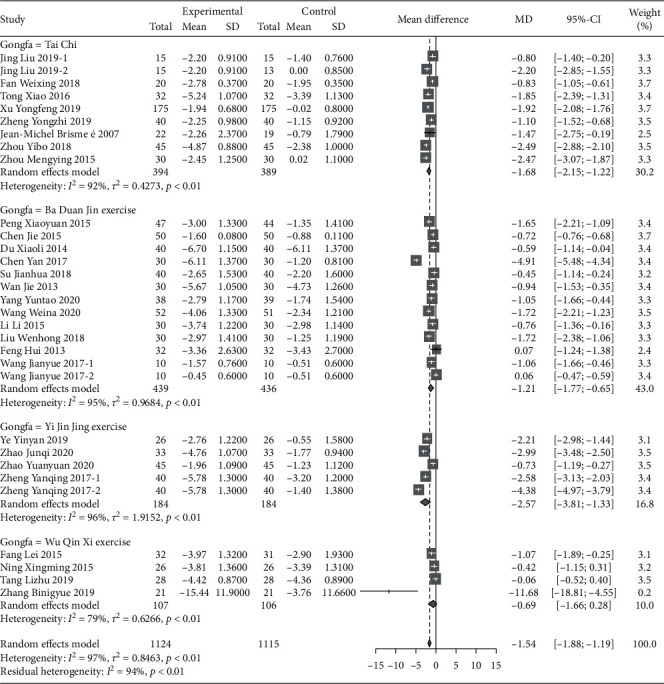
Subgroup analysis of different VAS comparisons.

**Figure 5:**
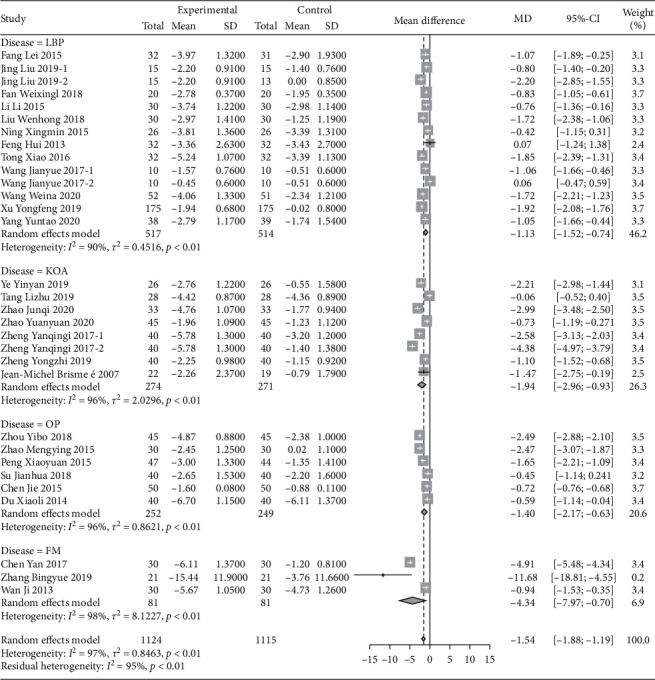
Subgroup analysis of the comparison of pain VAS scores of different diseases.

A total of 28 RCT trials, which included 2239 patients, were analyzed. VAS scores of patients with musculoskeletal pain who intervened in the Chinese traditional exercise group were significantly lower than those in the control group [MD = −1.54, 95% CI (−1.88, −1.19), *P* < 0.01], as shown in Figure 3. According to the subgroup analysis based on the types of traditional exercises, Tai Chi, Ba Duan Jin exercises, and Yi Jin Jing exercise all presented significant therapeutic effects on the VAS score of patients with musculoskeletal pain. Among them, the Yi Jin Jing exercise showed the best therapeutic effect (Figure 4). The analysis between Wu Qin Xi exercise subgroups showed no statistical difference. Two studies [66, 67] treated the disease for low back pain within the subgroup, and one study [58] focused on knee osteoarthritis. According to the subgroup analysis based on the types of musculoskeletal pain diseases, the results show that traditional Chinese exercise therapy provided significant positive therapeutic effects on knee osteoarthritis, chronic low back pain, osteoporotic pain, and fibromyalgia. Among them, the treatment of osteoporosis pain provided the best results, as shown in Figure 5.

#### 3.2.2. Oswestry Dysfunction Index (ODI) Score

**Figure 6:**
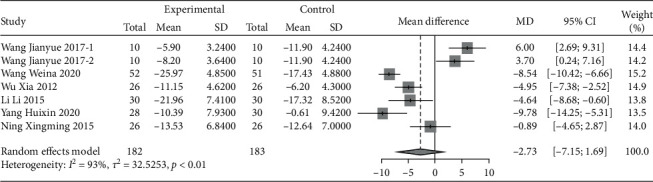
Meta-analysis of the comparison of ODI scores between the traditional Chinese exercise group and the control group.

Six studies, which included a total of 365 patients, were analyzed. The ODI scores of patients with musculoskeletal pain with the intervention of traditional Chinese exercise were significantly lower than those of the control group [MD = −2.73, 95% CI (−7.15, −1.69), *P* < 0.01], as shown in Figure 6. The six studies were all related to low back pain. According to the subgroup analysis of the exercise type, the intervention effect of Yi Jin Jing exercise [56] was significantly better than that of Tai Chi [45, 48–50] and Wu Qin Xi exercise [67].

#### 3.2.3. Western Ontario and McMaster University Osteoarthritis Index (WOMAC)

**Figure 7:**
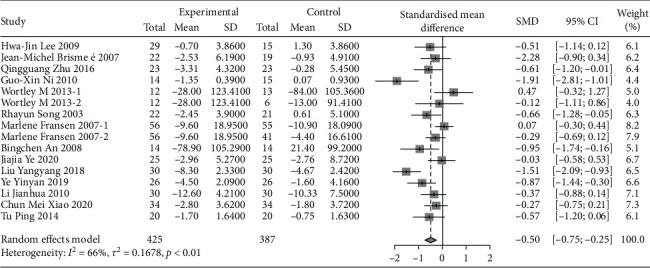
Meta-analysis of WOMAC pain comparison between the traditional Chinese exercise group and the control group.

**Figure 8:**
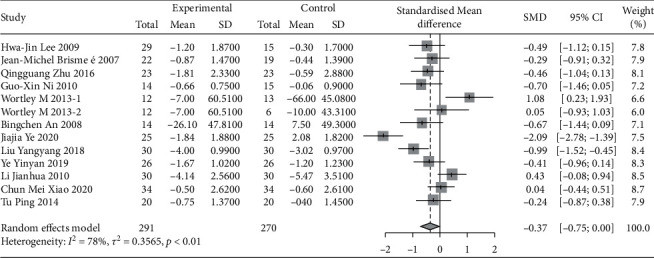
Meta-analysis of WOMAC stiffness comparison between the traditional Chinese exercise group and the control group.

**Figure 9:**
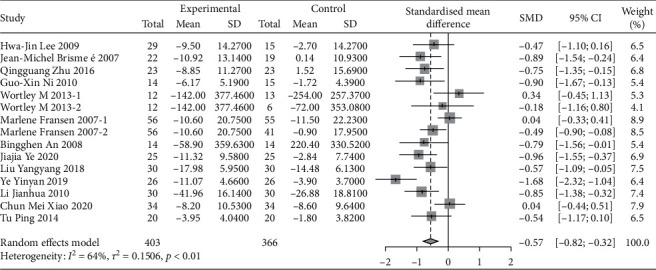
Meta-analysis of WOMAC physical function comparison between traditional Chinese exercise group and control group.

A total of 17 studies were evaluated using WOMAC. Due to the large difference in the mean between the study groups, SMD combined statistics were selected. The results showed that traditional Chinese exercise was used to treat knee arthritis and was able to reduce WOMAC pain [SMD = −0.50, 95% CI (−0.75, −0.25), *P* < 0.01], relieve joint WOMAC stiffness [SMD = −0.37, 95% CI (−0.75, −0.00), *P* < 0.01], and improve dysfunction [SMD = −0.57, 95% CI (−0.82, −0.32), *P* < 0.01]. All results were statistically significant, as shown in Figures 7–9. Besides, according to the type of exercise method, a subgroup analysis of WOMAC showed that the Ba Duan Jin exercise intervention was significantly better than other exercise methods in reducing pain and improving dysfunction. In contrast, the Yi Jin Jing exercise was superior to other exercises in improving joint stiffness.

#### 3.2.4. Sensitivity Analysis

The sensitivity analyses of VAS exclude four studies with higher weights [38, 48, 49, 66], and the heterogeneity did not change significantly. The sensitivity analyses of ODI eliminate the two studies with higher weights [55, 56], and heterogeneity has changed significantly (*I*^2^ = 66%, *T*^2^ = 6.4961, *P* = 0.03), but the result does not change. Further analysis was done on two studies, and its heterogeneous sources may be related to the younger age of research subjects. Sensitivity analysis for WOMAC pain, excluding a study [46], reduced to moderate heterogeneity. Sensitivity analyses for WOMAC stiffness, WOMAC Physical Function, and Liu [47], Ye et al. [40], and Ye et al.'s [57] research studies were excluded. According to the Cochrane handbook [24], the three indicators are all reduced to moderate heterogeneity. The source of the articles' heterogeneity may relate to the low age of the included patients and the imbalance of the gender ratio between men and women.

#### 3.2.5. Publication Bias

**Figure 10:**
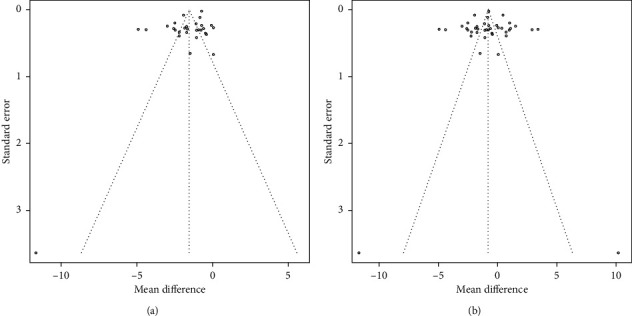
The funnel plots of VAS.

The results show that VAS was biased (*t* = −3.2289, df = 29, *P* *=* 0.003082), and the use of the trimming filling method to adjust the published bias resulted in statistically significant differences (*P* < 0.001), as seen in Figure 10. WOMAC index of the three-funnel chart and metabias regression analysis shows no publication bias. Furthermore, the reliability of this study was high.

## 4. Discussion

Traditional Chinese exercises have been widely used in clinics to prevent various diseases, improve the quality of life, and increase happiness [70]. The results of this study showed that Tai Chi, Yi Jin Jing exercise, and Ba Duan Jin exercise in traditional Chinese significantly reduced the pain score of musculoskeletal diseases consistently across multiple meta-analysis studies [71–74]. Among musculoskeletal diseases, traditional Chinese exercise had the most significant impact on osteoporosis pain, which may be related to the cause of pain in each subordinate disease. Chronic low back pain, knee osteoarthritis, and fibromyalgia syndrome may be caused by noninfectious inflammation of joints. Therefore, proper traditional Chinese exercises are expected to relieve muscle spasms, prevent muscle strength decline [75], and relieve pain caused by inflammation.

Severe bone pain caused by osteoporosis, mainly due to the high bone turnover rate and increased bone resorption, leads to the destruction of bone microstructure. However, traditional Chinese exercise can improve bone biomechanics, regulating bone growth and development, promoting local blood circulation in bone, increasing bone cell activity, and reducing bone turnover and pain [52]. In the traditional Chinese exercise, Yi Jin Jing belongs to the group of high intensity of musculoskeletal strengthening exercises, and it is better than other traditional Chinese exercises for improving musculoskeletal diseases. However, there are only a few studies in this area. In analyzing the low back pain indicator ODI, traditional Chinese exercises play a significant therapeutic role. However, most studies are Tai Chi interventions on this indicator and could not distinguish the dominant types of exercises for the treatment of low back pain. For the intervention of knee arthritis, Tai Chi and Ba Duan Jin exercise had significantly positive effects on pain and stiffness in patients with knee arthritis, consistently with the results of previous studies by Xie et al. [74, 76–78]. However, the therapeutic effect of the Yi Jin Jing exercise was only noted for joint stiffness and dysfunction, while the Wu Qin Xi exercise did not present a significant therapeutic value on all the evaluated indicators.

These results can be further analyzed from the specific characteristics of each exercise. In the process of these exercises, for changes in body posture and shifting of the gravity center, Tai Chi and Ba Duan Jin exercise apply more pressure to the knee joint than the Yi Jin Jing exercise. Also, the sports characteristics of Tai Chi focus more on the coordination of various parts of the body, the speed, strength, flexibility, and others [79]. Therefore, Tai Chi is used to improving the balance ability in the elderly and preventing falls [80, 81]. The Ba Duan Jin exercise comprises movements of holding the knees with hands, swinging the body, and moving the center of gravity, which is beneficial for multiangle and large-scale movement of the knee joint. Yi Jin Jing exercise mainly focuses on strengthening muscles and bones [82], and only a few movements are aimed at the knee joint. Although studies have explored the effect of Yi Jin Jing exercise on the knee joint, the current research [83, 84] only has focused on the dysfunction and activity of the knee joint. However, we cannot ignore the possibility that the available literature may be scarce and include biased results. Wu Qin Xi exercise did not have a significant therapeutic effect on musculoskeletal pain. This may be related to the design purpose of the Wu Qin Xi exercise, which was to imitate the five movements of the tiger, deer, bear, ape, and bird to stretch and strengthen their body to prevent diseases [85]. Therefore, the intensity of intervention on the musculoskeletal part of the body is not as focused as traditional Chinese exercises.

Nonetheless, there are some limitations in our meta-analysis. Firstly, several uncontrollable variables of the patients, such as age and disease, may affect the results. Secondly, a few articles included in this review did not explicitly report the procedures for random sequence generation, allocation concealment, and the blinding of assessors. Hence, selection and detection biases may have affected the validity of our results. Finally, most of the participants in many research studies were elderly, which may contribute to a biased result. In the future, more RCTs that focused on the incidence of musculoskeletal pain in a specific age group may be needed to corroborate our results further.

## 5. Conclusion

The traditional Chinese therapeutic exercises provided a more significant improvement effect on VAS, ODI, and WOMAC scores, with the Yi Jin Jing exercise being the best exercise in changing VAS. The Ba Duan Jin exercise was the most impactful in treating joint stiffness. However, additional large-sample studies with strict designs are needed to prove the therapeutic effects of different traditional Chinese exercises in CMP patients.

## Figures and Tables

**Table 1:** Basic characteristics of the included studies.

Study	Example (person)		Average age (y)		Mode of intervention		Disease	Ending indicator
	Experimental group	Control group	Experimental group	Control group	Test group	Control group		
Lee et al. [25]	29	15	70.2 ± 4.8	66.9 ± 6.0	Tai Chi	No intervention	Knee osteoarthritis	③
Liu et al. [26]	15	15	58.13 ± 5.38	58.4 ± 5.08	Tai Chi	Core stability training	Low back pain	①
Liu et al. [26]	15	13	58.13 ± 5.38	60.67 ± 2.58	Tai Chi	No rehabilitation plan	Low back pain	①
Zhu et al. [27]	23	23	64.61 ± 3.40	64.53 ± 3.43	Tai Chi	No rehabilitation plan	Knee osteoarthritis	③
Brismée et al. [28]	22	19	70.8 ± 9.8	68.8 ± 8.9	Tai Chi	Health education	Knee osteoarthritis	③
Fransen et al. [29]	56	55	70.8 ± 6.3	69.6 ± 6.1	Tai Chi	Spa course	Knee osteoarthritis	③
Fransen et al. [29]	56	41	70.8 ± 6.3	70.0 ± 6.3	Tai Chi	None	Knee osteoarthritis	③
Wortley et al. [30]	15	9	69.5 ± 6.7	70.5 ± 5.0	Tai Chi	Open motion chain resistance training	Knee osteoarthritis	③
Wortley et al. [30]	15	9	68.1 ± 5.3	70.5 ± 5.0	Tai Chi	None	Knee osteoarthritis	③
Song et al. [31]	22	21	64.8 ± 6.0	62.5 ± 5.6	Tai Chi	Conventional treatment	Knee osteoarthritis	③
Xu and Zhang [32]	83	85	21.05 ± 1.15	21.34 ± 2.06	Tai Chi	None	Low back pain	①
Zhao et al. [33]	30	30	58.8 ± 3.2	60.1 ± 2.8	Tai Chi	None	Osteoporosis	①
Zheng et al. [34]	40	40	66.25 ± 6.01	67.10 ± 6.51	Tai Chi	Drug treatment	Knee osteoarthritis	①
Zhou et al. [35]	45	45	71.86	72.25	Tai Chi	Drug treatment	Osteoporosis	①
Xu and Tang [36]	15	14	62.89 ± 2.79	63.47 ± 2.85	Tai Chi	Education and stretching sessions and	Knee osteoarthritis	③
Fan [37]	20	20	55.7 ± 8.64	56.4 ± 9.12	Tai Chi	Moxibustion	Low back pain	①
Tong et al. [38]	32	32	32.60 ± 6.46	32.66 ± 6.53	Tai Chi	Sleep in a hard bed	Low back pain	①
An et al. [39]	14	14	65.4 ± 8.2	64.6 ± 6.7	Ba Duan Jin exercise	No intervention	Knee osteoarthritis	③
Ye et al. [40]	25	25	64.48 ± 7.81	63.08 ± 3.65	Ba Duan Jin exercise	Physical exercise	Knee osteoarthritis	③
Yang et al. [41]	40	40	54.20 ± 13.30	53.94 ± 13.42	Ba Duan Jin exercise	Regular massage combined with waist and dorsal muscle exercise	Low back pain	①
Chen [42]	50	50	61.2 ± 4.9	60.8 ± 5.8	Ba Duan Jin exercise	Chinese medicinal diet	Osteoporosis	①
Chen et al. [43]	30	30	63.57 ± 4.71	62.27 ± 4.66	Ba Duan Jin exercise	Rehabilitation physiotherapy, health education, strict sleeper rigid bed, drug treatment	Osteoporosis	①
Du and Zhao [44]	40	40			Ba Duan Jin exercise	Sodium alan phosphate + calcium agent osteoporosis	Osteoporosis	①
Li and Feng [45]	30	30	45.77 ± 2.11	46.38 ± 2.33	Ba Duan Jin exercise	SET therapy	Low back pain	①②
Liu et al. [46]	30	30	26.6 ± 0.8	27.3 ± 1.1	Ba Duan Jin exercise	General physiotherapy	Low back pain	①
Liu [47]	30	30	53.26 ± 3.87	53.47 ± 3.61	Ba Duan Jin exercise	Regular care	Knee osteoarthritis	③
Wu [48]	26	26	55.92 ± 9.25	56.46 ± 9.13	Ba Duan Jin exercise	Intermediate electrotherapy	Low back pain	①
Wang and Zhao [49]	52	51	46.51 ± 4.31	45.97 ± 4.22	Ba Duan Jin exercise	Rehabilitation training	Low back pain	①②
Wang et al. [50]	10	10	17.10 ± 1.20	17.60 ± 1.08	Ba Duan Jin exercise	Pure wormwood box moxibustion + eight brocades	Low back pain	①②
Wang et al. [50]	10	10	16.90 ± 1.10	17.60 ± 1.08	Ba Duan Jin exercise	Auricular-plaster therapy + pure wormwood box moxibustion	Low back pain	①②
Wan et al. [51]	30	30	40.97 ± 11.62	42.87 ± 10.87	Ba Duan Jin exercise	Manipulation maneuver	Fibromyalgia syndrome	①
Su and Deng [52]	40	40	58.93 ± 4.01	59.12 ± 3.88	Ba Duan Jin exercise	Take medicine	Osteoporosis	①
Peng et al. [53]	47	44	68.49 ± 4.68	69.67 ± 4.36	Ba Duan Jin exercise	Take medicine	Osteoporosis	①
Pang et al. [54]	32	32	46.33 ± 9.46	47.25 ± 8.43	Ba Duan Jin exercise	Take medicine	Low back pain	①
Zheng and Cheng [55]	29	30	57.01 ± 5.59	58.63 ± 5.07	Ba Duan Jin exercise	Acupuncture treatment	Knee osteoarthritis	①③
Yang [56]	28	30	21.52 ± 1.95	20.41 ± 2.09	Yi Jin Jing exercise	None	Low back pain	①
Ye et al. [57]	26	26	60.83 ± 9.52	61.80 ± 8.26	Yi Jin Jing exercise	Perkin ontology and balance training	Knee osteoarthritis	①③
Zhao and Zhang [58]	33	33	73.84 ± 4.69	72.94 ± 5.97	Yi Jin Jing exercise	Western medicine treatment	Knee osteoarthritis	①③
Zhao et al. [59]	45	45	64.00 ± 8.97	61.00 ± 7.52	Yi Jin Jing exercise	Inject sodium glassate	Knee osteoarthritis	①
Zhen et al. [60]	40	40			Yi Jin Jing exercise	Moxibustion	Knee osteoarthritis	①③
Zhen et al. [60]	40	40			Yi Jin Jing exercise	Western medicine treatment	Knee osteoarthritis	①③
Wu and Lu [61]	45	45	54.56 ± 10.07	58.02 ± 7.93	Yi Jin Jing exercise	Massage and intra-articular ozone injection	Knee osteoarthritis	①③
Li et al. [62]	30	30			Yi Jin Jing exercise	Massage manipulation therapy	Knee osteoarthritis	③
Li et al. [63]	62	67	69.5 ± 4.8	69.3 ± 4.5	Yi Jin Jing exercise	Physiotherapy	Knee osteoarthritis	①③
Xiao et al. [64]	34	34	70.7 ± 9.36	70.2 ± 10.35	Wu Qin Xi exercise	Rehabilitation treatment	Knee osteoarthritis	③
Zhang et al. [65]	21	21	52.90 ± 10.57	56.19 ± 10.88	Wu Qin Xi exercise	Oral amitriptyline hydrochloride tablets	Fibromyalgia syndrome	①
Lei et al. [66]	32	31	52.91 ± 15.80	53.88 ± 14.17	Wu Qin Xi exercise	Rehabilitation gymnastics	Low back pain	①
Ning et al. [67]	26	28	40.73 ± 11.52	42.13 ± 11.18	Wu Qin Xi exercise	Nuclear myocardial force training and five poultry exercises	Low back pain	①②
Tang et al. [68]	30	30	60.36 ± 4.73	59.86 ± 5.92	Wu Qin Xi exercise	Massage combined with isokinetic training	Knee osteoarthritis	①
Ping and Liao [69]	20	20			Wu Qin Xi exercise	Standing exercise	Knee osteoarthritis	③

Note: ① VAS; ② ODI; ③ WOMAC.

## Data Availability

The data used to support the findings of this study are available on request from the corresponding author.
